# Hydrophilic and lipophilic radiopharmaceuticals as tracers in pharmaceutical development: *In vitro *– *In vivo *studies

**DOI:** 10.1186/1471-2385-5-5

**Published:** 2005-10-18

**Authors:** Mariella Terán, Eduardo Savio, Andrea Paolino, Malcolm Frier

**Affiliations:** 1 Cátedra de Radioquímica – Facultad de Química – Universidad de la República. Montevideo, Uruguay; 2 Radiopharmacy Unit. Queen's Medical Centre, Nottingham University. Nottingham, UK

## Abstract

**Background:**

Scintigraphic studies have been performed to assess the release, both *in vitro *and *in vivo*, of radiotracers from tablet formulations. Four different tracers with differing physicochemical characteristics have been evaluated to assess their suitability as models for drug delivery.

**Methods:**

In-vitro disintegration and dissolution studies have been performed at pH 1, 4 and 7. In-vivo studies have been performed by scintigraphic imaging in healthy volunteers. Two hydrophilic tracers, (^99m^Tc-DTPA) and (^99m^Tc-MDP), and two lipophilic tracers, (^99m^Tc-ECD) and (^99m^Tc-MIBI), were used as drug models.

**Results:**

Dissolution and disintegration profiles, differed depending on the drug model chosen. *In vitro *dissolution velocity constants indicated a probable retention of the radiotracer in the formulation. *In vivo *disintegration velocity constants showed important variability for each radiopharmaceutical. Pearson statistical test showed no correlation between *in vitro *drug release, and *in vivo *behaviour, for ^99m^Tc-DTPA, ^99m^Tc-ECD and ^99m^Tc-MIBI. High correlation coefficients were found for ^99m^Tc-MDP not only for *in vitro dissolution *and disintegration studies but also for *in vivo *scintigraphic studies.

**Conclusion:**

Scintigraphic studies have made a significant contribution to the development of drug delivery systems. It is essential, however, to choose the appropriate radiotracers as models of drug behaviour. This study has demonstrated significant differences in release patterns, depending on the model chosen. It is likely that each formulation would require the development of a specific model, rather than being able to use a generic drug model on the basis of its physicochemical characteristics.

## Background

Development of new drug formulations requires the performance of extensive studies, both in the laboratory, and *in vivo*, in animals and in volunteers. *In vitro *studies can be very expensive but costs are even higher when *in vivo *stages are reached. Methodology that can generate relevant information but shorten the preformulation phases means important savings in economic, human and time terms [1-9]. Gamma scintigraphy provides rapid, complementary information that often cannot be obtained by other methodologies. It has been successfully used during development stages of feasibility studies and in determining specific parameters of the final product. The information obtained by scintigraphy gives support during investigation and development, and also complements the development of registration dossiers and marketing publicity. The incorporation of a radiopharmaceutical into a drug formulation allows determination of the biodistribution kinetics and the release sites [10-14]. It is very important to choose the proper radionuclide, often ^99m^Tc (technetium), as this has optimal characteristics of half-life and energy, allowing images to obtain with high efficiency and low doses [15,16].

Many studies to validate the methodology mentioned above have already been undertaken [17,18], but, in general within these studies, radiopharmaceuticals are only rarely used as drug surrogates. Radiopharmaceuticals were primarily developed for diagnostic purposes in nuclear medicine, most of them being intended for intravenous administration. In order to optimise the usage of radiopharmaceuticals in the development of pharmaceutical drug-delivery systems, the behaviour of these tracers following administration by other routes must be validated. Stability and *in vitro *studies of different radiopharmaceuticals have been previously reported by our group [17], with the aim of creating a database of radiotracers or model drugs with known physicochemical properties to be used as in pharmaceutical dosage development, particularly of tablet formulations.

Provided that the tablet formulation can be radiolabelled with a suitable gamma emitter without altering its characteristics, imaging with a gamma camera can be used to monitor *in vivo *transit and dispersion of the tablet, and give some indication of deposition and absorption of the drug. The aim of this work is to assess *in vivo *and *in vitro behaviour *of a tablet formulation using scintigraphic studies, to examine the way in which radiopharmaceuticals model the release of drugs, using four different tracers with different physicochemical characteristics. Based on this assessment, the correlation between in-vitro and in-vivo behaviour could be investigated.

The characterised radiopharmaceuticals were ^99m^Tc-diethylenetriamine-pentaacetic acid (^99m^Tc-DTPA), ^99m^Tc-ethyl cysteinate dimer (^99m^Tc-ECD), ^99m^Tc-methylene diphosphonate (^99m^Tc-MDP) and ^99m^Tc-sestamibi (^99m^Tc-MIBI) [17]. They were incorporated into the tablets during wet granulation. Disintegration profiles were assessed *in vitro *in dissolution vessels and *in vivo *by scintigraphic imaging in healthy volunteers. *In vitro *dissolution studies were performed for tablets containing the above-mentioned radiotracers

## Methods

### Radiopharmaceuticals labelling and control

The radiopharmaceuticals ^99m^Tc-DTPA, ^99m^Tc-ECD, ^99m^Tc-MDP and ^99m^Tc-MIBI were obtained by labelling commercial kits with ^99m^TcO_4_^- ^from a ^99^Mo /^99m^Tc generator (Technonuclear). Radiochemical purity testing of the ^99m^Tc-DTPA complex was performed by chromatographic analysis on Whatman N° 1 paper with propanone and sodium chloride solution (0.9%) as developing solvents.

^99m^Tc-ECD radiochemical purity was studied by chromatography on Whatman N°1 paper with a methanol/water (85/15) solvent and by HPLC (Shimadzu LC-10 AS) using a Partisphere C18 (Whatman) column as the stationary phase. The mobile phases were phosphate buffer 0.0125 M pH 2.5 (A), and ethanol 100% (B). Solvent program was, at time = 0 min, 100 % A, and at time = 10 min, 70% A, 30% B. The flow rate was set at 2 mL min^-1^.

The radiochemical purity of ^99m^Tc-MDP was determined by chromatography on Whatman N°1 paper using propanone and sodium chloride 0.9 % as developing solvents.

The ^99m^Tc-MIBI complex was assessed by HPLC (Shimadzu LC-10 AS) using a Partisphere C18 (Whatman) column 12.5 cm as stationary phase. The mobile phases were methanol (A), and ammonium sulphate 0.05 M (B). Solvents program was at time = 0 min, 20 % A, and at time = 5 min, 95% A. The flow rate was set at 2 mL min^-1^.

### Tablet preparation

Tablets were prepared by wet granulation using lactose and starch as diluents, PVP K30 (ISP Corp) as granulating agent, Ac-Di-Sol (FMC Biopolymer) as disintigrant and magnesium stearate as lubricant. The exipients were passed through a No. 16 sieve (16 meshes per centimetre) and mixed by a geometric method. After compression, tablets were assessed for weight (target weight 400 mg) and radioactivity content (target activity 18.5 MBq). Activity was measured in an ionisation chamber dose calibrator (Capintec CRC 25).

Tracer stability was verified as in a previous communication [18] both during tablet preparation and in dissolution and disintegration studies.

### *In vitro *studies

Dissolution tests were carried out in a Vankel USP Type II apparatus, 900 mL of dissolution medium was used at three different pH values comprising pH 1 (0.1 M hydrochloric acid), pH4 (0.016 M acetic acid/sodium acetate) and pH 7 (water). Temperature was maintained at 37°C, and a rotation speed of 50 r.p.m. used. Samples of 2 mL were withdrawn through cellulose acetate membrane filters (0.22 μm) at 1, 2, 3, 5, 10, 15 and 30 minutes and assessed for radioactivity in a solid scintillation counter (Ortec-Maestro MCB1). Corrections were applied for volume, decay and counting efficiency. Curves of log % non-dissolved vs. time were plotted.

Disintegration profiles were recorded concurrently with the dissolution procedure by placing the vessels in front of a circular field of view gamma camera (Dyna (Pyker 4/15) equipped with a low energy, high resolution collimator, operating with a 20% window centred on 140 Kev. Serial images were recorded at 1 frame/min with a matrix of 128 × 128 without acquisition zoom. Three regions of interest were delineated, one on the tablet, one on the dissolution medium and the third on the external field to determine the background [19]. Net activity was calculated by subtracting mean background pixel counts from each pixel in the selected region of interest. No attenuation correction was necessary.

### *In vivo *studies

Studies were performed in four healthy volunteers (2 men and 2 women) mean weight 60 Kg, mean age 38 years. They were non smokers and were not receiving any medication. Written informed consent was obtained from all subjects. They were not allowed to consume alcohol during the study period or in the preceding 24 hours. The protocol was in accordance with the Declaration of Helsinki guidelines for ethics in research and with resolutions XXIX and XXXV of the World Medical Assembly.

Volunteers fasted for 6 hours prior to the study. Tablets were administered with 200 mL of still mineral water. Volunteers stood erect during the acquisition process in front of the detector. Urine samples were collected at 30 and 120 minutes post ingestion [22,23].

Serial images were recorded during the 30 minute period after administration at 2 frames / min using a rectangular field view gamma camera (Sophy). Later static images were recorded 2 hours after tablet intake.

Three regions of interest were delineated, one on the stomach, one on the intestines and a third on a region outside the body perimeter to determine the background. Net activity was quantified by subtracting mean background pixel counts from each pixel in the selected region of interest. Gamma ray attenuation was quantified by calculating the geometric means of count rates in paired anterior and posterior planar views.

Scintigraphic studies enabled the determination of tablet transit characteristics within the gastrointestinal tract specifically in the stomach. Curves of Log % non-disintegrated vs. time were plotted [24,25].

### Data processing

Disintegration velocity constant of the tablets was k_d_, which corresponded to the slope of the linear regression in first order kinetics [26]:

log [1- Q_t_/Q_∞_] = k_d_t / 2.303

Where

Q_t _the amount of activity of the tracer disintegrated at time *t *present in the region of interest.

Q_∞ _the maximum amount of activity measured in the region of interest.

Disintegration velocity constants in the stomach and the constant of appearance in small intestine were determined in a similar way, considering Q_t _the amount of activity of the tracer disintegrated at time *t *present in the region of interest and Q_∞ _the total amount of activity of the region at the end of the study [27].

## Results and Discussion

Radiotracers were incorporated during the wet granulation process of tablet preparation and appropriate controls of quality were applied as previously described [17]. All the radiopharmaceuticals were produced with radiochemical purities higher than 95 %, in accordance with manufacturers' specifications.

Disintegration velocity constants in the gastrointestinal tract were determined by *in vivo *scintigraphic studies, specifically in stomach and small intestine for the different radiopharmaceuticals evaluated as tracers.

### *In vitro *results

#### ^99m^Tc-MIBI

Dissolution velocity constants for tablets containing this lipophilic tracer showed no significant variation across the whole pH range studied and the values were very similar to disintegration velocity constants determined under the same conditions (Table 1).

**Table 1:** In vitro dissolution and disintegration constants (p 0.05)

Radiopharmaceutical	Dissolution velocity Constant (min^-1^) (n = 6)	Disintegration velocity Constant (min^-1^) (n = 6)
^99m^Tc-MIBI	(0.05 ± 0.02) all pH range	(0.04 ± 0.01) all pH range
^99m^Tc-ECD	(0.040 ± 0.005) all pH range	(0.05 ± 0.01) (pH 1) (0.09 ± 0.01) (pH4 and 7)
^99m^Tc-MDP	(0.020 ± 0.005) all pH range	(0.20 ± 0.04) (pH 1 and 7) (0.040 ± 0.005) (pH 4)
^99m^Tc-DTPA	(0.06 ± 0.01) (pH1and 7) (0.11 ± 0.02) (pH 4)	0.011 ± 0.002 (pH1and 7) 0.080 ± 0.006 (pH 4)

The Pearson statistical test was used to quantify correlation within both series of data. Pearson's correlation coefficient *r *is always between -1 and 1 indicating that the points are near a negative or a positive slope respectively. The closer the *r *value is to -1 or 1 the higher the correlation of the series is. [27]

Dissolution and disintegration velocity constants (*r *values) were compared using this test. Correlation coefficients are shown in Table 3.

**Table 3:** *In vitro *dissolution – disintegration Pearson correlation coefficient

pH	^99m ^Tc MIBI	^99m ^Tc ECD	^99m ^Tc MDP	^99m ^Tc DTPA
1	0.75	0.99	0.68	0.99
4	0.77	0.78	0.90	0.99
7	0.50	0.94	0.68	0.99

They were not so high indicating a probable retention of the radiotracer in the formulation.

#### ^99m^Tc-ECD

This lipophilic radiopharmaceutical demonstrated identical dissolution velocity constants across the whole pH range studied, while disintegration velocity constants increased with pH (Table 1).

Pearson correlation coefficients are shown in Table 3. A high correlation was found.

#### ^99m^Tc-MDP

*In vitro *dissolution velocity constants did not show significant variation across the whole pH range while *in vitro *disintegration velocity constants increased with pH (Table 1). *In vitro *correlation coefficients were high at all pH values. (Table 3)

#### ^99m^Tc-DTPA

*In vitro *dissolution and disintegration data showed similar patterns but the values were different (Table 1). In this case at pH 1 and 7 both disintegration and dissolution velocity constants were lower than those at pH 4. The profiles were similar in both series of data and Pearson correlation was very high at all pH ranges studied. (Table 3)

### *In vivo *results

#### ^99m^Tc- MIBI

**Figure 1:**
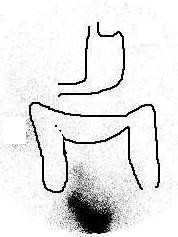
^99m^Tc-MIBI placebo tablet scintigraphic image 2 hours after drug intake for one volunteer.

No significant variability within volunteers was observed in stomach disintegration velocity constants. Small intestine appearance constant showed similar behaviour but different values and did not represent significant differences within volunteers (Table 2). Two hours after administration there was no significant uptake in any organ except urinary bladder (Figure 1). Pearson Test was used to compare *in vitro *– *in vivo *disintegration velocity constants (Table 4) assuming pH 1 for stomach and pH 4 for small intestine no correlation was found. When Pearson test was used for *in vivo *disintegration velocity constants and *in vitro *dissolution ones, (Table 5) correlation coefficient was again low for stomach and a bit higher for small intestine, but in neither case was correlation considered to be established.

**Table 2:** In vivo gastric transit constants (p 0.05)

Radio pharmaceutical	Stomach disintegration velocity Constant (min^-1^) (n = 4)	Small intestine appearance Constant (min^-1^) (n = 4)
^9m^Tc-MIBI	(0.016 ± 0.006)	(0.13 ± 0.02)
^99m^Tc-ECD	From (0.7 ± 0.1) to (0.010 ± 0.002)	(0.02 ± 0.01)
^99m^Tc-MDP	(0.007 ± 0.002)	(0.037 ± 0.006)
^99m^Tc-DTPA	From (0.13 ± 0.03) to (0.07 ± 0.01)	From (0.004 ± 0.001) to (0.08 ± 0.01)

**Table 4:** *In vitro*-*In vivo *disintegration velocity constants Pearson correlation coefficient.

pH	^99m ^Tc MIBI	^99m ^Tc ECD	^99m ^Tc MDP	^99m ^Tc DTPA
1	0.29	0.44	0.29	0.8
4	0.21	0.29	0.08	0.12

**Table 5:** *In vitro *dissolution – *in vivo *disintegration Pearson correlation coefficient

pH	^99m ^Tc MIBI	^99m ^Tc ECD	^99m ^Tc MDP	^99m ^Tc DTPA
1	0.13	0.08	0.68	0.07
4	0.51	0.69	0.59	0.12

#### ^99m ^Tc ECD

**Figure 2:**
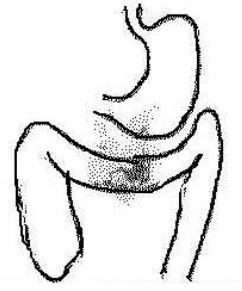
Scintigraphic image 2 hours after drug intake for one volunteer ^99m^Tc-ECD placebo tablets.

In this particular case there was a substantial inter subject variability with coefficient variations during the 30-minute study period ranging from 14 to 95 % (Table 2). Small intestine appearance velocity constants were more homogeneous and late images 2 hours after administration showed liver uptake (Figure 2).

Correlation coefficient was not significant when *in vitro *disintegration – *in vivo *appearance velocity constants were compared (Table 4).

When Pearson correlation was quantified between *in vivo *disintegration velocity constants and *in vitro *dissolution ones (Table 5) as indicated for ^99m^Tc-MIBI, there was no correlation at pH 1 (stomach) and it was low at pH 4 (small intestine).

#### ^99m ^Tc- MDP

**Figure 3:**
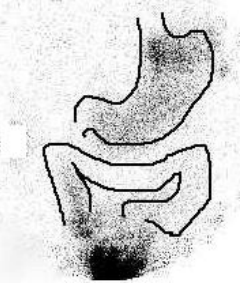
Scintigraphic image 2 hours after drug intake for one ^99m^Tc-MDP placebo tablets in one volunteer.

No significant variations within volunteers were observed in stomach disintegration velocity constants. Small intestine appearance constant showed similar behaviour but different values and did not represent significant differences within volunteers (Table 2). Two hours after administration there was no significant uptake in any organ except urinary bladder (Figure 3). Pearson Test was used to compare *in vitro *– *in vivo *disintegration velocity constants (Table 4). In this case correlation was negligible. When Pearson Test was used for *in vivo *disintegration velocity constants and *in vitro *dissolution ones, (Table 5) correlation coefficients had the highest values found in this study.

#### ^99m ^Tc- DTPA

**Figure 4:**
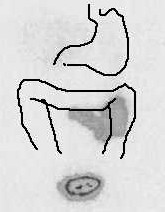
Scintigraphic image 2 hours after drug intake for one volunteer ^99m^Tc-DTPA placebo tablets.

Significant variations within volunteers were observed in stomach disintegration velocity constants and small intestine appearance constants (Table 2). Two hours after administration there was no significant uptake in any organ except urinary bladder (Figure 4). Pearson Test was used to compare *in vitro *– *in vivo *disintegration velocity constants (Table 4).

In this case correlation was negligible for pH 4 and higher for pH 1. When Pearson Test was used for *in vivo *disintegration velocity constants and *in vitro *dissolution ones, (Table 5) correlation was negligible for the entire gastrointestinal tract.

A good correlation was found between *in vitro *disintegration and dissolution velocity constants of tablets containing each of the radiopharmaceuticals used as radiotracer.

As scintigraphic studies give information of a physical process, tablets containing the same components, but different tracers, might be expected to have a similar behavior in all cases. As tracers are present in low concentrations (lower than 10^-9 ^M), it is unlikely that they are responsible for the observed differences in the disintegration constants. It is possible that the tracers are bound to different components within the tablet, which disperse at different rates, which would be a likely explanation. Although the dissolution profiles might be expected to differ depending on the model drug used, the same would not be expected of the disintegration profile. This suggests that the measured disintegration profile can depend on the model chosen.

The radiopharmaceutical ^99m^Tc ECD showed absorption in the gastrointestinal tract. This is not a desirable characteristic and makes it an unsuitable tracer for this type of study. It was probably the main factor giving rise to the greater inter individual variability disintegration velocity constants observed when *in vivo *studies were performed for ^99m^Tc-ECD formulations. This fact was not observed with the other radiotracers under the same conditions of study.

Despite the physicochemical differences between ^99m^Tc-MIBI and ^99m^Tc-DTPA, both tracers presented very low correlation coefficients when *in vitro *dissolution – *in vivo *disintegration velocity constants were compared throughout the whole gastrointestinal tract (Table 3).

Only ^99m ^Tc-MDP showed high correlation coefficients both *in vitro *between dissolution and disintegration, and between *in vitro *dissolution and *in vivo *disintegration.

## Conclusion

Even though ^99m^Tc-ECD, ^99m^Tc-MIBI and ^99m^Tc-DTPA showed high *in vitro *correlation between dissolution and disintegration, statistical tests revealed that none of them was an adequate predictor of *in vivo *performance for this particular tablet formulation, for any region of the gastrointestinal tract.

The hydrophilic radiotracer ^99m^Tc-MDP was the only radiopharmaceutical suitable as a drug model for this particular tablet formulation in the prediction of its *in vivo *behavior.

The method is an interesting tool especially at early stages of pharmaceutical formulation development when different formulations are being chosen, but may not have adequate sensitivity to discriminate between formulation variables.

Careful choice of drug model, together with substantial *in vitro *validation is essential in order to reduce *in vivo *studies and make significant savings of human and financial resources.

## Competing interests

The author(s) declare that they have no competing interests.

## Authors' contributions

Mrs. Mariella Terán carried out all the experimental work and together with Dr. Eduardo Savio made substantial contributions to conception, design, analysis and data interpretation. They also gave their final approval of the version to be published. Mrs. Andrea Paolino contributed to data acquisition, analysis and interpretation. Dr. Malcolm Frier was involved in revising it critically for important intellectual content.

## Pre-publication history

The pre-publication history for this paper can be accessed here:


